# The Passing Year

**Published:** 1893-12

**Authors:** 


### THE PASSING YEAR.

This number will close the work of the year and finish Volume XIV. of this journal.

The retrospect of a year's work invariably brings with it a commingling of pleasure and regret, in which both feelings are so closely intermingled that it is difficult, at times, to know which predominates. While this is probably true of every life and every occupation, it must, from the nature of the duties, be peculiarly the experience of those connected with journalistic work.

The labor here is never completed, but there are resting-places which afford time to review results, take stock, as it were, of its mental and scientific value, and draw a balance between the good and the bad, the talent that builds for the future and that which is ephemeral in character.

Reverting to the work peculiarly our own, it is with some degree of pride that we can point to twelve numbers of excellent material, much of it of a high order of merit from a scientific standard. The aim has been, from its inception, to make the journal the organ of the best thought of the dental profession, admitting nothing to its pages of a trivial character. This was regarded as in consonance with that higher standard which should be the effort of the best intelligence in dentistry to cultivate.

It would be untrue to say that our ideal of a dental periodical has been reached. The time has not yet arrived for this to appear. It will come when dentists are fully alive to their responsibilities as professional men, and are prepared to throw off the shackles of trade influence and the journals connected therewith. We have a great admiration for the energy displayed by our contemporaries, and regard the work performed as valuable, but it would be quite as reasonable to expect an outsider to represent the family, in all its peculiar and intimate relations, as to regard it possible for the so-called trade journals to represent the inner life of a large body of professional men.

We do not propose to argue this delicate point, but desire to impress our readers with the importance of special work in the direction of a better professional life. It is the one great weakness of dentistry. The indifference is sometimes appalling, and is, perhaps, more apparent to those obliged to work in the literature of our calling than to others not so favorably situated. It, however, shows itself everywhere, and the past year has demonstrated, as never before, the necessity for real missionary work in this direction.

To do this effectively there is no better agent than the monthly journal independent in all its relations, but to make this of value it must receive the cordial support of the entire body of dentists. Their first duty is to those of their own household.

As far as this journal is concerned we have no cause for serious complaint; indeed the outlook is most encouraging. The aid we have received from individuals and societies has been of the greatest value, and we desire to extend, in this general way, our earnest thanks for their co-operation in the work.

While thus encouraged, it is felt that the leaven of a higher standard is but slowly making its way into the thoughts and practice of the majority, and to this large number we must go, meet them in their daily work, cultivate by the evening fireside in many homes a taste for the scientific side of the profession they have adopted.

If this be the great need of the hour, is it asking too much for each one to constitute himself a special and self-appointed committee to rouse his immediate professional circle to greater activity in every direction? This is to be the principal work of the coming years.

The past is finished. The year 1893 has been one of great activity in certain directions, but whether this means a better professional life or otherwise is still a doubtful question. We have not much faith in spasmodic efforts. They are sure to be followed by periods of weakness. The only true advance is made slowly, securing every step before another is taken. In this way will each passing year show a gain and a material improvement in professional thought and work.

